# Physical Decay of Cities

**Published:** 1887-07

**Authors:** 


### PHYSICAL DECAY OF CITIES.

Lord Brabazon, in a paper in the *Nineteenth Century*, on the "Decay of Bodily Strength in Towns," says with reference to England: "If we could isolate the city and prevent all intermarriage with the country, the degeneration in the physique of the inhabitants of the former, would probably be so marked as to horrify the public. It is only necessary," he says, "for an intelligent man or woman to walk through the slums of our great towns" to recognize the substantial facts in the case.

What is true in these respects, in other countries, is true of ours, and especially so of the crowded sections of the city of New York.

Let any one visit one of our rural districts, for example, of New Jersey, and take notice of the strong, healthy, good natured children he sees upon either hand, and landing at any one of our down town ferries, on his return, let him observe the little, pale, slender, weezel-faced youngsters that crowd our sidewalks, looking as if it were an effort requiring all their strength to live, and he will need no argument or strong array of statistics to convince him that something should be done, and that too, at once, toward improving the physical condition of this numerous growing population. Good air, clean water and healthy food, and enough of them, are cheaper, in the end, than pest houses, infirmaries and insane asylums.

"Almost every nation in Europe," says the writer, "except ourselves, has established * * a system of compulsory physical training" in its schools "and spends large sums of money in strengthening the bodies and nerves of its future citizens." In Germany, besides its gymnastic training, each child at school is supplied with a sound midday meal, and if the parent cannot pay for it the State does. In the Swedish schools there is a physical drill accompanied by music. France has taken the hint from Germany, and since 1870 is paying some attention to building up the bodily strength of her youth."

### LACTATED FOOD

for infants and invalids is coming largely into use on its merits, after a multitude of tests in the nursery and the sick-room.

That manufactured by Wells, Richardson & Co., of Burlington, Vt., is highly spoken of.

